# Combining Cystatin C and Creatinine Increases eGFR Slope Accuracy in ADPKD

**DOI:** 10.1016/j.ekir.2026.107008

**Published:** 2026-08-06

**Authors:** Thomas Bais, Abdulfataah A.A. Mohamed, A. Noruzi, Muhammad Umar, Bella A. Binoy, M. Salih, Esther Meijer, Ron T. Gansevoort

**Affiliations:** 1 Division of Nephrology, Department of Internal Medicine, University Medical Center, Groningen, University of Groningen, Groningen, The Netherlands; 2 Department of Clinical Pharmacy and Pharmacology, University Medical Center Groningen, University of Groningen, Groningen, The Netherlands; 3 Division of Nephrology and Transplantation, Department of Internal Medicine, Erasmus Medical Center Rotterdam, The Netherlands

**Keywords:** ADPKD, estimated glomerular filtration rate, trial design

## Abstract

**Introduction:**

In autosomal dominant polycystic kidney disease (ADPKD) trials, creatinine-based estimated glomerular filtration rate (GFR, eGFR_cr_) is a key end point; however, variability in creatinine measurements may affect reliability. We compared routine eGFR_cr_ to eGFR derived from batch-measured creatinine, cystatin C (eGFR_cys_), or both (eGFR_cr-cys_) using biobanked samples, against iothalamate-measured GFR (mGFR).

**Methods:**

In the 260 patients with ADPKD from the DIPAK observational cohort, we compared 2021 Chronic Kidney Disease (CKD)-Epidemiology Collaboration (CKD-EPI)–based eGFR based on the following: (i) routine clinical creatinine, (ii) batch-measured creatinine, and (iii) batch-measured cystatin C to mGFR. Creatinine and cystatin batch measurements were performed on the same day to minimize variability. Cross-sectional performance was evaluated by bias (eGFR − mGFR), precision, accuracy (P30/P10), and CKD stage agreement. Longitudinal agreement between mGFR and eGFR slopes (ml/min per 1.73 m^2^/yr) was assessed using concordance correlation coefficients (CCC). Sample size requirements for hypothetical trials were calculated based on slope variability.

**Results:**

Cross-sectional performance of batch-measured eGFR_cr_ was largely similar to routine eGFR_cr_. eGFR_cys_ and eGFR_cr-cys_ showed greater bias than eGFR_cr_; eGFR_cr-cys_ showed the highest precision. Longitudinally, batch-measured eGFR_cr-cys_ slopes correlated more strongly with mGFR decline than eGFR_cr_ (CCC: 0.68; 95% confidence interval [CI]: 0.59–0.76] vs. 0.58 [95% CI: 0.47–0.67]; *P* = 0.02). eGFR_cr-cys_ slopes exhibited lower SDs than eGFR_cr_ and eGFR_cys_. This reduction in slope variability translated to sample size reductions of 37% to 38% for clinical trials.

**Conclusion:**

The use of eGFR_cr-cys_ enhances precision and reduces eGFR slope variability. Using combined creatinine-cystatin C estimates may optimize trial efficiency and design in ADPKD.

## Graphical abstract

**Figure:**
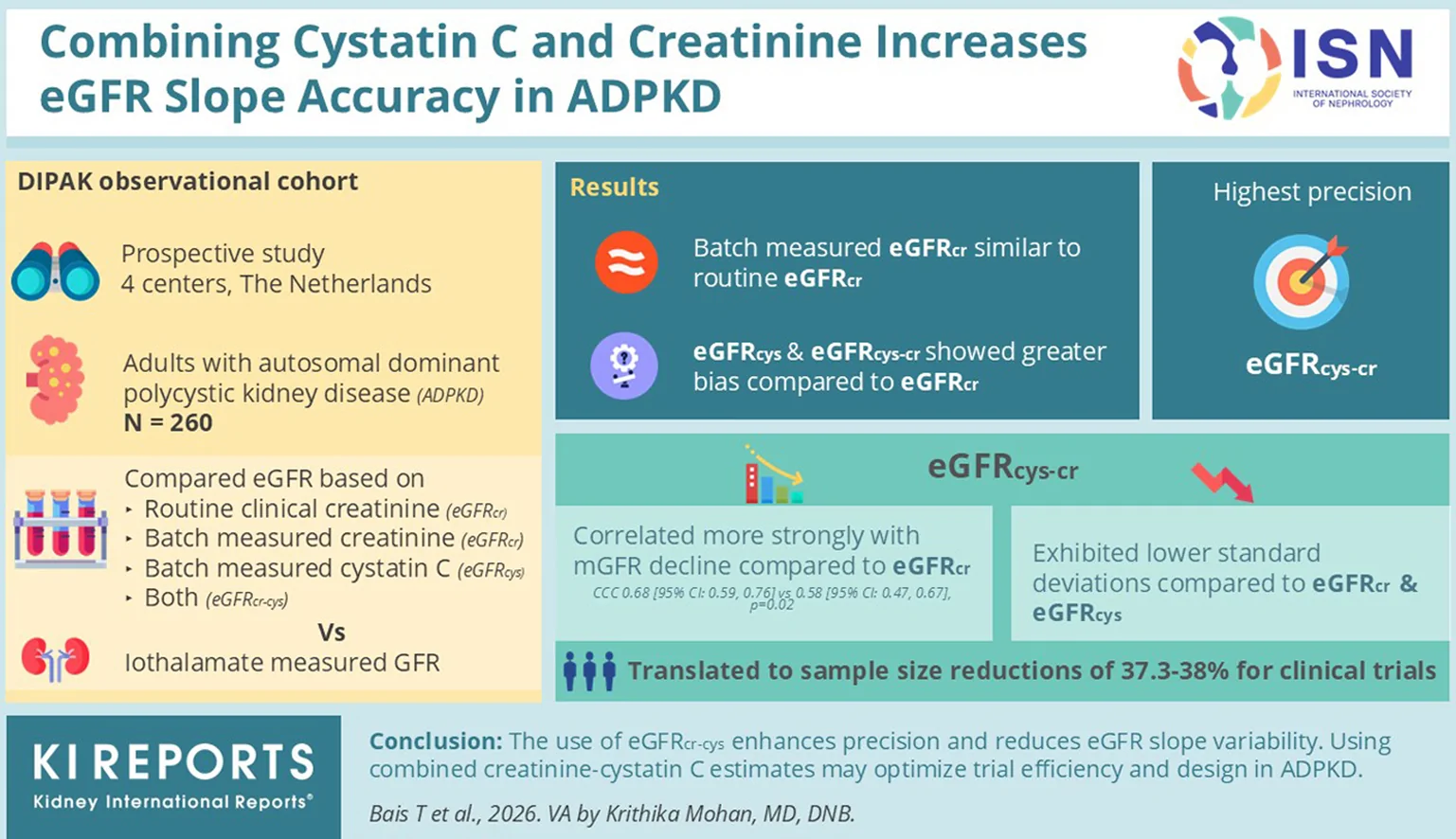

ADPKD is the most common hereditary kidney disorder, with an estimated prevalence of 4:10,000 individuals.^1^ The disease is characterized by kidney cyst formation, kidney growth, and progressive loss of kidney function.^2^^,^^3^ Majority of patients with ADPKD ultimately develop kidney failure that requires kidney replacement therapy.^3^ ADPKD accounts for approximately 10% of all patients on kidney replacement therapy.^1^^,^^4^, ^5^, ^6^, ^7^ There is only 1 treatment (tolvaptan) that has been shown to reduce the rate of eGFR decline to some extent. Because of the unmet need, there is an ongoing search for novel treatments to delay progression to kidney failure. In clinical trials that investigate the efficacy of these treatments, the rate of GFR decline is used as an end point.^8^

In these trials, change in GFR can be assessed in several ways. mGFR, using exogenous filtration markers, provides an accurate assessment of kidney function; however, its routine use is limited by costs, invasiveness, and resource requirements.^9^^,^^10^ Therefore, in clinical practice and research, eGFR is often used, derived from endogenous filtration markers such as creatinine (eGFR_cr_), cystatin C (eGFR_cys_), or both (eGFR_cr-cys_), using various equations. Creatinine, a breakdown product of creatine primarily found in skeletal muscle, is influenced by muscle mass.^10^^,^^11^ Cystatin C, a protein produced by all nucleated cells, offers an alternative endogenous filtration marker that is less affected by muscle mass; but can be influenced by other factors, such as obesity, inflammation, thyroid dysfunction, smoking, and corticosteroid use.^12^^,^^13^

In clinical trials, eGFR decline is typically assessed using plasma creatinine or cystatin C (or both), obtained from routine laboratory measurements during the study. However, the accuracy of this approach may be limited by intra- and interlaboratory assay variability.^14^ An alternative strategy is to biobank samples at trial visits and measure creatinine or cystatin C in a single batch at a central laboratory after trial completion, thereby minimizing variability, and potentially improving GFR precision. In addition, although many ADPKD clinical trials rely on creatinine-based eGFR, the combined use of creatinine and cystatin C for GFR estimation may reduce the influence of non-GFR determinants on eGFR variability, thereby improving the precision and reliability of eGFR slope estimates.

The aim of this study was to evaluate the reliability of eGFR slope estimates derived from 2 approaches—routine clinical creatinine measurements and batch-measured creatinine from biobanked samples. In addition, we aimed to assess whether the concordance between eGFR slope and mGFR slope differs when eGFR is derived from annual routine creatinine measurements versus 3-yearly study-visit measurements, and whether adding cystatin C improves this concordance. Finally, we determined how these different eGFR methods influence sample size requirements for clinical trials in ADPKD.

## Methods

### Study Setting and Population

We analyzed data from the ongoing Developing Intervention Strategies to Halt Progression of ADPKD (DIPAK) study, a prospective observational cohort study that includes adult patients (aged ≥ 18 years) with ADPKD recruited from 4 University Medical Centers (UMCs) in the Netherlands from 2013 onward. None of the patients were treated with lanreotide. The diagnosis of ADPKD was made through genetic testing or the Pei-Ravine criteria.^15^ Patients were excluded if they had primary kidney diseases other than ADPKD, systemic conditions impacting kidney function (such as diabetes mellitus), or take medications known to negatively change kidney function (like lithium).

For this study, we used data of 260 patients seen at the UMC Groningen (UMCG), who have mGFR assessments every 3 years, accompanied by simultaneous eGFR_cr_ in routine laboratory testing, and biobanking of plasma and urine samples. In addition, annual eGFR_cr_ measurements have been obtained through routine laboratory testing at the various hospitals where patients were monitored for routine clinical care. These annual measurements were performed in the local hospital laboratories on samples taken on the same day as the clinical visit. In addition, all participating laboratories used IDMS-traceable creatinine assays. An overview of all GFR measurements and estimates used in this study, including the method in which it was determined, is presented in Table 1. The study complies with the International Conference on Harmonization Good Clinical Practice guidelines and received approval from the institutional review board of UMCG (METc 2013/040). All participants provided written informed consent before inclusion.

**Table 1:** Summary of GFR estimates used in this study

Estimate	Method
Cross-sectional (baseline only)	
mGFR	Measured GFR using radioactive tracers (Supplementary Methods)
eGFR_cr-routine_	Creatinine-based eGFR from routine study visit assays
eGFR_cr-batch_	Creatinine-based eGFR from centrally analyzed stored plasma
eGFR_cys-batch_	Cystatin C–based eGFR from centrally analyzed stored plasma
eGFR_cr-cys-batch_	Creatinine–cystatin C-based eGFR from centrally analyzed stored plasma
Longitudinal	
mGFR slope	Measured GFR slope from 3-yearly study visits
eGFR slope_cr-routine-corresponding_	Creatinine-based eGFR slope from routine 3-yearly study visit assays
eGFR slope_cr-routine-annual_	Creatinine-based eGFR slope from annual clinical care measurements (including from outside laboratories/hospitals)
eGFR slope_cr-batch_	Creatinine-based eGFR slope using centrally batch-analyzed stored plasma samples from 3-yearly study visits
eGFR slope_cys-batch_	Cystatin C–based eGFR slope using centrally batch-analyzed stored plasma samples from 3-yearly study visits
eGFR slope_cr-cys-batch_	Creatinine and cystatin C–based eGFR slope using centrally batch-analyzed stored plasma samples from 3-yearly study visits

eGFR, estimated GFR; GFR, glomerular filtration rate; mGFR, measured GFR.

### Measurement of GFR

Simultaneous infusion of radiolabeled ^125^I-iothalamate and ^131^I-hippuran was used to determine mGFR, as previously described.^16^ Detailed mGFR methodology is presented in the Supplementary Methods. mGFR was indexed for body surface area calculated using the Mosteller equation.^17^

### Measurement of Plasma Creatinine and Cystatin C

Lithium-heparin plasma samples were collected during the 3-yearly study visits at the UMCG, at the same time points as mGFR, and stored at −80 °C. Creatinine and cystatin C were measured in these stored plasma samples in a single centralized laboratory batch after long-term storage. Plasma creatinine was measured in the central clinical chemistry laboratory of the UMCG, using an IDMS-traceable enzymatic assay (Roche Modular Analyzer; Roche Diagnostics, Mannheim, Germany). Cystatin C, which had not been measured previously, was quantified in 1 batch with validated particle-enhanced turbidimetric immunoassays (Roche Diagnostics, Mannheim, Germany), using the same frozen samples. Biobanked samples used in this study were thawed for the first time to avoid potential influence of repeated thaw-freeze cycles on creatinine or cystatin C levels.

For the primary analyses, eGFR_cr_, eGFR_cys_, and eGFR_cr-cys_ were respectively calculated using the 2021 creatinine-based CKD-EPI,^18^ 2012 cystatin-based CKD-EPI,^19^ and the 2021 creatinine-cystatin based CKD-EPI^18^ equations.

### Clinical and Radiological Data Collection

Data on sex, age, CKD stage, height-adjusted total kidney volume (TKV), urinary albumin-to-creatinine ratio, body mass index, smoking status, and plasma high-sensitivity C-reactive protein were collected for the subgroup analyses. TKV was assessed using an automated stereological method applied to T2-weighted magnetic resonance imaging scans. Height-adjusted TKV was calculated by dividing TKV by height, and indexed for age to calculate the Mayo Imaging Classification. Genotyping was previously described.^20^ Blood pressure was recorded as the mean of 3 automated measurements.

### Statistical Analysis

eGFR_cr_ calculated from routine creatinine measurements obtained in the clinic (eGFR_cr-routine_), were compared with the mGFR at the same time points. In addition, comparisons were made using eGFR values derived from creatinine (eGFR_cr-batch_) and cystatin C (eGFR_cys-batch_) measured in 1 batch using biobanked samples. For baseline mGFR measurements, the cross-sectional performance of eGFR was evaluated using performance metrics, including bias, precision, accuracy, and agreement between eGFR and mGFR categories. Bias was defined as the mean difference between eGFR and mGFR (eGFR − mGFR), with positive values indicating an overestimation of mGFR. Precision was determined as the SD of the difference between eGFR and mGFR. Accuracy was assessed by the percentage of participants whose eGFR fell within 30% and 10% of their mGFR (P30/P10). Agreement was assessed between eGFR and mGFR CKD categories (< 15, 15–29, 30–44, 45–59, 60–89 and ≥ 90 ml/min per 1.73 m^2^). For these performance metrics, 95% CIs were obtained by bootstrapping with 1000 iterations.

In addition to cross-sectional analyses, the longitudinal performance of eGFR estimates as a surrogate for mGFR decline over time was assessed. mGFR and eGFR slopes (ml/min per 1.73 m^2^/yr) were determined for each patient using regression of mGFR or eGFR against time until maximum follow-up, using the following 3 approaches: (i) annual routine creatinine measurements (eGFR slope_cr-routine-annual_), (ii) routine creatinine values matched to corresponding mGFR time points (eGFR slope_cr-routine-corresponding_), and (iii) batch-measured creatinine values from biobanked samples matched to mGFR time points (eGFR slope_cr-batch_). For eGFR slope_cr-routine-annual_, slope calculations were performed with annually performed eGFR measurements obtained from various hospitals and laboratories. eGFR slope_cr-routine-corresponding_ were calculated using eGFR measured in the UMCG laboratory at study visits (every 3 years). In addition, eGFR slopes were calculated using batch-measured cystatin C (eGFR slope_cys-batch_) and both batch-measured creatinine and cystatin (eGFR slope_cr-cys-batch_) at matched mGFR time points. To ensure alignment of timeframes, eGFR values obtained beyond the last available mGFR measurement were excluded from the analyses. The agreement between mGFR and eGFR slopes was assessed using the CCC,^21^ which unlike Pearson’s *r,* is sensitive to consistent bias and therefore, a measure of both precision and accuracy. Bland-Altman plots were used to illustrate the mean error margin of the difference (eGFR slope − mGFR slope) and ±1.96 SD limits of agreement.

Exploratory subgroup analyses for bias in slope estimates were conducted based on sex, age, CKD stage, height-adjusted TKV, urinary albumin-to-creatinine ratio, body mass index, smoking status, and high-sensitivity C-reactive protein levels. For continuous variables, subgroups were dichotomized as values ≤ or > the median. For the sensitivity analyses, cross-sectional and longitudinal analyses were repeated with alternative eGFR equations, including the 2009 creatinine-based CKD-EPI^22^ (without race coefficient), the 2021 creatinine-based European Kidney Function Consortium (EKFC) equation,^23^ the 2023 cystatin-based EKFC equation,^24^ and the 2012 CKD-EPI and 2023 EKFC equations^19^^,^^24^ incorporating both markers.

To evaluate the variability and implications for sample size determination in clinical trials assessing kidney function decline in ADPKD, we compared GFR slopes of the following 5 different estimation methods: eGFR slope_cr-routine-annual_, eGFR slope_cr-routine-corresponding_, eGFR slope_cr-batch_, eGFR slope_cys-batch_, and eGFR slope_cr-cys-batch_. For this approach, we selected a subset of 99 patients who would typically be included in clinical ADPKD trials (aged ≤ 55 years, Mayo Imaging Classification 1C/1D/1E, and GFR ≥ 30 ml/min per 1.73 m^2^) and calculated their GFR slopes during the first 3 years of follow-up (a typical duration for interventional trials in ADPKD). We calculated required sample sizes for hypothesized treatment effects on eGFR slope of −20%, −25%, and −30%, assuming a typical 2-arm randomized controlled trial design with 80% power, a significance level (α) of 0.05, 1:1 allocation, and 20% dropout rate. The required sample sizes were derived for each method to illustrate how measurement frequency and variability influence trial efficiency.

Statistical analysis was performed using R version^25^ 4.4.2 and RStudio^26^ version 2024.04.2+764. Normally distributed variables were presented as mean ± SD and nonnormally distributed variables as median (interquartile range). The choice of statistical test was guided by the distribution of the data (normal/nonnormal) and the dependency of observations (independent or paired/longitudinal). Differences between subgroups were evaluated using *t*-tests (for 2 groups) or 1-way analysis of variance (for ≥ 3 groups) for normally distributed quantitative data, with paired tests used for dependent observations. For nonnormally distributed data, the Mann-Whitney U test (2 groups) or Kruskal-Wallis test (≥ 3 groups) was applied for independent data, whereas the Wilcoxon signed-rank test or Friedman test was used for dependent data. Categorical data were analyzed using the Chi-square test or McNemar’s test for independent and dependent observations, respectively. Hypothesis tests were 2-tailed, and *P*-values < 0.05 were considered statistically significant.

## Results

### Cohort Characteristics

A total of 260 patients were included (44% male), with a mean age of 45.1 ± 12.0 years and mGFR of 70.2 ± 28.2 ml/min per 1.73 m^2^ at baseline. Baseline characteristics are displayed in Table 2. Among 189 patients (73%) with multiple available mGFRs, 49 had 2 mGFR measurements (3.8 ± 1.4 years of follow-up), 78 had 3 (6.1 ± 0.8 years), and 62 had 4 (9.0 ± 0.4 years). Patients with an increasing number of mGFR measurements (and longer follow-up) were more often male, were more often in higher risk Mayo Imaging Classification (1C/1D/1E), and had a higher mGFR at baseline. The mean mGFR slope during follow-up was −3.01 ± 2.04 ml/min per 1.73 m^2^/yr. Out of 260 patients, 85 discontinued the study because of initiation of kidney replacement therapy (*n* = 38), withdrawn consent (*n* = 35), lost to follow-up (*n* = 7) or death (*n* = 5).

**Table 2:** Baseline characteristics and follow-up duration of the study cohort

Variable	Number of mGFR measurements				
	Overall (*N* = 260)	1 (*n* = 71)	2 (*n* = 49)	3 (*n* = 78)	4 (*n* = 62)
Sex, male	114 (43.8)	26 (36.6)	12 (24.5)	41 (52.6)	35 (56.5)
Age, yrs	45.1 ± 12.0	43.5 ± 13.2	47.1 ± 11.6	46.9 ± 11.5	43.1 ± 10.9
BMI, kg/m^2^	26.7 ± 5.0	27.9 ± 6.7	26.2 ± 4.7	26.4 ± 4.1	26.1 ± 3.8
SBP, mm Hg	128.0 ± 11.6	126.0 ± 12.1	130.0 ± 11.7	127.7 ± 12.2	129.4 ± 10.0
Ethnicity					
-Asian	4 (1.5)	1 (1.4)	2 (4.1)	0 (0.0)	1 (1.6)
-Caucasian	253 (97.3)	70 (98.6)	46 (93.9)	77 (98.7)	60 (96.8)
-Turkish	2 (0.8)	0 (0.0)	1 (2.0)	0 (0.0)	1 (1.6)
-Other	1 (0.4)	0 (0.0)	0 (0.0)	1 (1.3)	0 (0.0)
Mayo imaging class					
-1A/1B	49 (18.8)	4 (5.6)	10 (20.4)	16 (20.5)	19 (30.6)
-1C	80 (30.8)	17 (23.9)	16 (32.7)	27 (34.6)	20 (32.3)
-1D/1E	84 (32.3)	13 (18.3)	19 (38.8)	31 (39.7)	21 (33.9)
-Atypical	7 (2.7)	2 (2.8)	2 (4.1)	2 (2.6)	1 (1.6)
-Not available	40 (15.4)	35 (49.3)	2 (4.1)	2 (2.6)	1 (1.6)
CKD stage					
-G1	70 (26.9)	25 (35.2)	9 (18.4)	13 (16.7)	23 (37.1)
-G2	74 (28.5)	10 (14.1)	11 (22.4)	24 (30.8)	29 (46.8)
-G3a	47 (18.1)	10 (14.1)	8 (16.3)	22 (28.2)	7 (11.3)
-G3b	43 (16.5)	9 (12.7)	14 (28.6)	17 (21.8)	3 (4.8)
-G4	25 (9.6)	16 (22.5)	7 (14.3)	2 (2.6)	0 (0.0)
-G5	1 (0.4)	1 (1.4)	0 (0.0)	0 (0.0)	0 (0.0)
mGFR, ml/min per 1.73 m^2^	70.2 ± 28.2	66.2 ± 34.4	61.4 ± 28.4	67.6 ± 23.2	84.6 ± 20.4
Follow-up duration, years	4.7 ± 3.4	-	3.8 ± 1.4	6.1 ± 0.8	9.0 ± 0.4

BMI, body mass index; CKD, chronic kidney disease; mGFR, measured glomerular filtration rate; SBP, systolic blood pressure.

Values are given as mean ± SD or *n* (%).

### Routine Versus Batch-Measured eGFR_cr_

**Figure 1:**
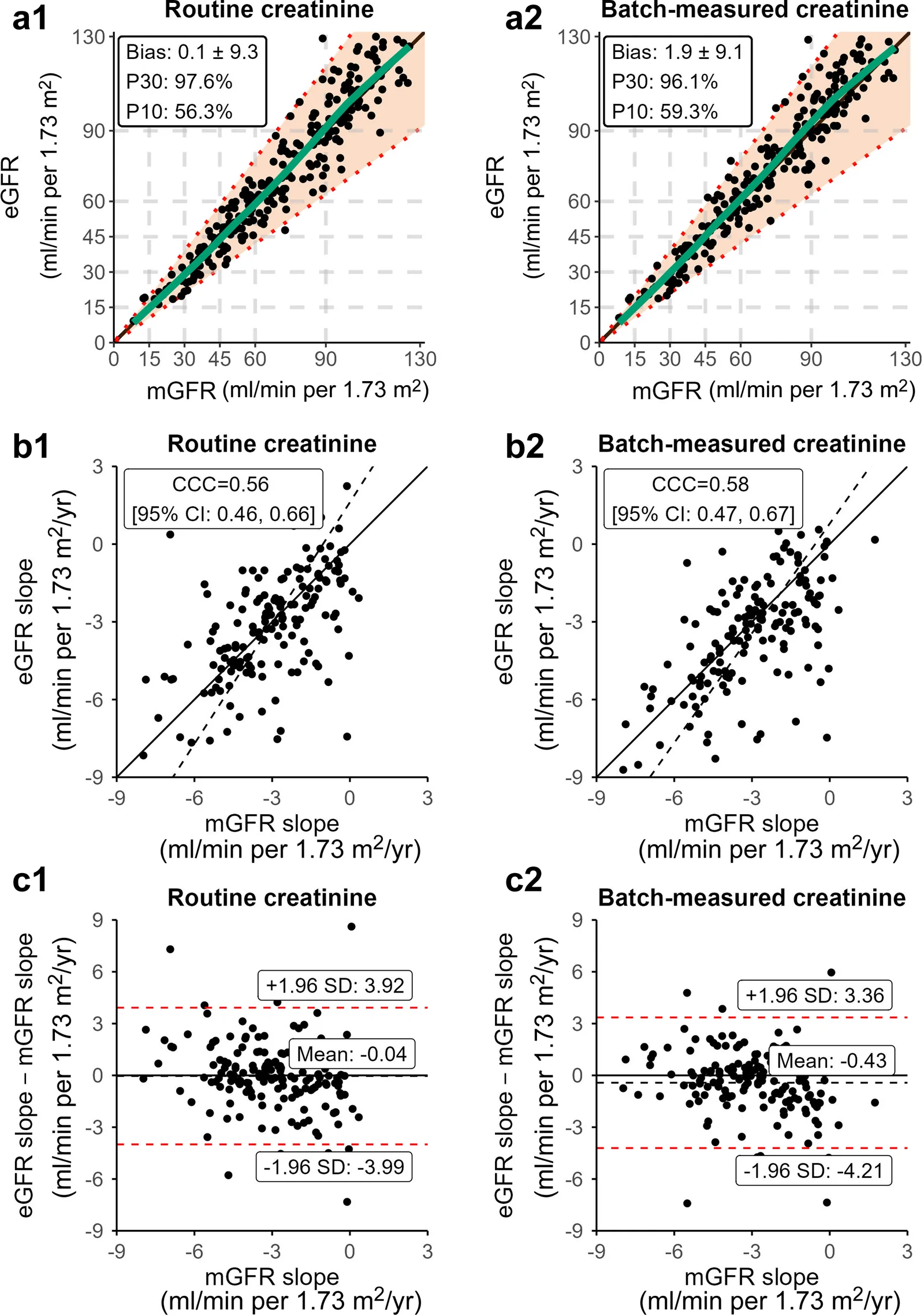
eGctional mGFR (a1 and a2) and GFR slope (b1–c2). a1 and a2: Baseline bias ± precision (eGFR − mGFR, ml/min per 1.73 m^2^) using routine (a1) and batch creatinine (a2). Black = identity; red dotted = P30 (±30%); gray dashed = CKD stages; green = LOESS (locally estimated scatterplot smoothing). b1 and b2: mGFR vs. eGFR slopes (routine b1; batch b2). Dotted = orthogonal regression; solid = identity. c1–c2: Bland–Altman plots of slopes (routine c1; batch c2). Black dotted = mean bias; red dotted = limits of agreement. CCC, concordance correlation coefficient; CI, confidence interval; CKD-EPI, Chronic Kidney Disease: Epidemiology Collaboration equation; eGFR, estimated GFR; GFR, glomerular filtration rate; mGFR, measured GFR.

For the cross-sectional analyses, 260 baseline mGFR assessments were available. The performance estimates for the baseline eGFR measurements are displayed in Figure 1 (panels a1 and a2) and Table 3. Mean bias for eGFR_cr-routine_ was 0.1 (95% CI: −1.3 to 1.0) ml/min per 1.73 m^2^, with a P30 and P10 of 98% (95% CI: 96–99) and 56% (95% CI: 50–62), respectively, and an agreement between mGFR and eGFR_cr-routine_ CKD stages of 76% (95% CI: 70–81). For eGFR_cr-batch,_ bias was 1.9 (95% CI: 0.8–3.1) ml/min per 1.73 m^2^, which was slightly larger than for eGFR_cr-routine_ (*P <* 0.001). Numbers for precision, P30, P10, and agreement were comparable with eGFR_cr-routine_. Results for alternative eGFR equations are included in Supplementary Table S1. Therefore, eGFR_cr-batch_ showed largely similar performance as eGFR_cr-routine_.

**Table 3:** Performance estimates for eGFR_cr-routine,_ eGFR_cr-batch_ and eGFR_cys-batch_ for cross-sectionally assessing mGFR, using various eGFR CKD-EPI equations

Variable	Bias (ml/min per 1.73 m^2^)	Precision (ml/min per 1.73 m^2^)	P30 (%)	P10 (%)	Agreement (%)
eGFR_cr-routine_ (CKD-EPI 2021)	0.1 (−1.0 to 1.3)	9.3 (8.2–10.4)	97.6 (95.5–99.2)	56.3 (50.2–62.3)	75.7 (70.4–81)
eGFR_cr-batch_ (CKD-EPI 2021)	1.9 (0.8–3.1)	9.1 (8.0–10.1)	96.1 (93.5–98.7)	59.3 (52.8–65.8)	77.5 (71.9–82.7)
eGFR_cys-batch_ (CKD-EPI 2012)	−5.5 (−6.6 to −4.4)	9.1 (8.2, 9.9)	97.4 (95.2, 99.1)	45.9 (39.8, 51.9)	63.6 (57.1, 69.7)
eGFR_cr-cys-batch_ (CKD-EPI 2021)	−1.5 (−2.4 to −0.5)	7.6 (6.7–8.5)	98.3 (96.5–99.6)	59.7 (53.2–65.8)	75.3 (69.7–81)

CKD-EPI, Chronic Kidney Disease Epidemiology Collaboration; eGFR, estimated glomerular filtration rate; EKFC, European Kidney Function Consortium; mGFR, measured glomerular filtration rate.

95% confidence intervals are presented in brackets.

The mean (95% CI) biases between routine and batch-measured plasma creatinine concentrations (routine − batch) at baseline, and at years 3, 6, and 9 were −2.2 (95% CI: −2.9 to −1.6), −2.3 (95% CI: −3.0 to −1.6), 0.02 (95% CI: −0.8 to 0.9), and −0.9 (95% CI: −2.1 to 0.3) μmol/l, respectively. Bias was modestly lower at year 6 than at the other time points (*P* < 0.001), while remaining comparable across the remaining measurements; suggesting that storage time did not meaningfully affect batch creatinine measurements.

For the longitudinal analyses (Figure 1, panels b1–c2), the correlation between eGFR slope_cr-routine-corresponding_ and mGFR slope (CCC = 0.56 [95% CI: 0.46–0.66]) was similar to that of eGFR slope_cr-batch_ (CCC = 0.58 [95% CI: 0.47–0.67]). Bland-Altman plots demonstrated similar limits of agreement for the batch-measured method, suggesting comparable precision. The CCC with mGFR slope for each eGFR slope method are presented in detail in Table 4 (results for the 2009 CKD-EPI and EKFC equations are included in Supplementary Table S2). Subgroup analysis for the bias between slopes (eGFR − mGFR slope) revealed a significant interaction for CKD category (*P* = 0.048), with early CKD stages (G1/G2) having a lower (more negative) eGFR slope_cr-routine_ than their mGFR slope (Supplementary Figure S1). For eGFR slope_cr-batch_ (Supplementary Figure S2), there was a significant interaction for age (*P* = 0.01), with younger patients (aged ≤ 46.5 years) having a slightly lower eGFR slope_cr-batch_ than their mGFR slope. In conclusion, the cross-sectional and longitudinal analyses suggested that eGFR using batch-measured creatinine values performs similarly to eGFR using creatinine values obtained routinely in the clinic.

**Table 4:** Summary of mGFR and eGFR slopes during follow-up with CCC (concordance correlation coefficient) estimates

Variable	Slope ± SD (ml/min per 1.73 m^2^/yr)	CCC (95% CI)
mGFR	−3.01 ± 2.04	-
eGFR_cr-routine-annual_ (CKD-EPI 2021)	−3.00 ± 1.97	0.49 (0.37–0.59)
eGFR_cr-routine-corresponding_ (CKD-EPI 2021)	−3.02 ± 2.43	0.56 (0.46–0.66)
eGFR_cr-batch_ (CKD-EPI 2021)	−3.39 ± 2.34	0.58 (0.47–0.67)
eGFR_cys-batch_ (CKD-EPI 2012)	−2.90 ± 2.05	0.61 (0.50–0.70)
eGFR_cr-cys-batch_ (CKD-EPI 2021)	−3.19 ± 2.02	0.68 (0.59–0.76)

CCC, concordance correlation coefficient; CI, confidence interval; CKD-EPI, Chronic Kidney Disease Epidemiology Collaboration formula; eGFR, estimated glomerular filtration rate; mGFR, measured glomerular filtration rate.

### Batch-Measured eGFR_cr_ Versus eGFR_cys_ and eGFR_cr-cys_

The cross-sectional and longitudinal performance of eGFR_cys-batch_ and eGFR_cr-cys-batch_ is displayed in Table 3 and Supplementary Figure S3. For eGFR_cys-batch_, cross-sectional bias was significantly larger than for eGFR_cr-batch_ (−5.5 [95% CI: −6.6 to −4.4] vs. 1.9 [95% CI: 0.8–3.1] ml/min per 1.73 m^2^, *P <* 0.001). Correspondingly, P10 for eGFR_cys-batch_ was significantly lower than eGFR_cr-batch_ (46% [95% CI: 40%–52%] vs. 59% [95% CI: 53%–66%], respectively, *P =* 0.006), and agreement was lower for eGFR_cys-batch_ (64% [95% CI: 57%–70%] vs. 78% [95% CI: 72%–83%], *P* = 0.001). Numbers for precision and P30 were similar. The correlation between eGFR slope_cr-batch_ and mGFR slope was comparable with that of eGFR slope_cys-batch_ (CCC = 0.61 [95% CI: 0.50–0.70], *P* = 0.9). Subgroup analysis for slope bias revealed no significant interactions for eGFR slope_cys-batch_ (Supplementary Figure S4).

For eGFR_cr-cys-batch_, the absolute bias was lower than for eGFR_cr-batch_ (−1.5 [95% CI: −2.4 to −0.5] vs. 1.9 [95% CI: 0.8–3.1] ml/min per 1.73 m^2^, *P* < 0.001). Notably, the SD of the bias was lower than eGFR_cr-batch_ (7.6 [95% CI: 6.7–8.5] vs. 9.1 [95% CI: 8.0–10.1] ml/min per 1.73 m^2^, *P* = 0.02), suggesting improved precision. Longitudinally, the correlation between eGFR slope_cr-cys-batch_ and mGFR slope (CCC = 0.68 [95% CI: 0.59–0.76]) was significantly higher than the correlations between mGFR slope and eGFR slope_cr-batch_ (*P* = 0.004) and eGFR slope_cys-batch_ (*P* = 0.001). For the CKD-EPI 2009 and EKFC equations, a similar trend was observed: equations incorporating both creatinine and cystatin C correlated more strongly with mGFR slope than those using either marker alone (Supplementary Table S2). GFR slope bias was influenced by CKD category (*P =* 0.03), with early CKD stages being associated with a lower (more negative) eGFR slope_cr-cys-batch_ slope than the mGFR slope (Supplementary Figure S5). Similarly, there was a trend (*P* = 0.06) toward a lower eGFR slope_cr-cys-batch_ in younger patients (aged ≤ 46.5 years), which corresponded to the findings for eGFR slope_cr-batch_.

**Figure 2:**
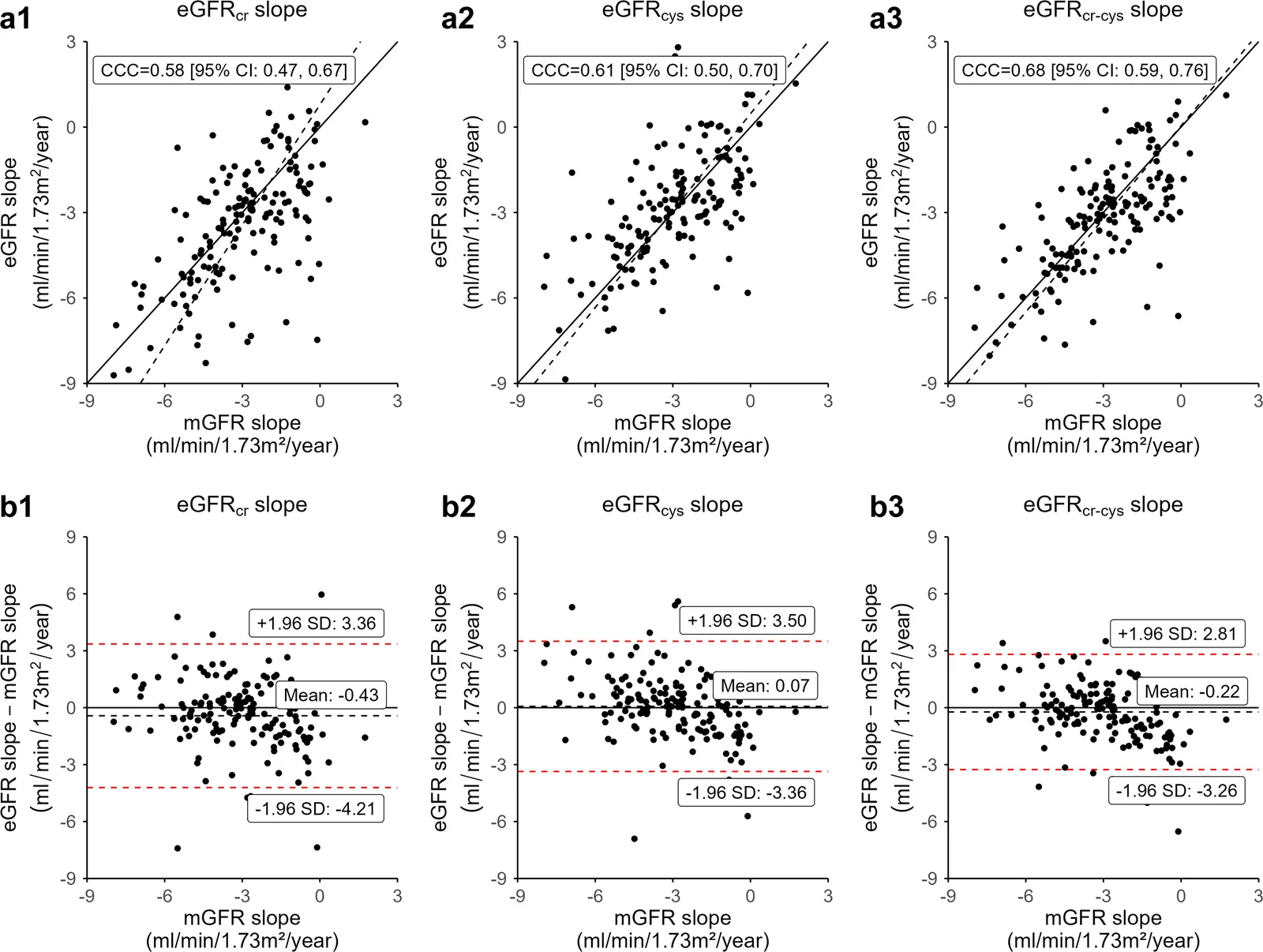
Longitudinal performance of eGFR slopes using batch-measured eGFR_cr_, eGFR_cys_, eGFR_cr-cys_ for tracking mGFR slopes. a1–a3: mGFR vs. eGFR slopes. Dotted = orthogonal regression; solid = identity. b1–b3: Bland–Altman plots of slopes. Black dotted = mean bias; red dotted = limits of agreement. CCC, concordance correlation coefficient; CI, confidence interval; eGFR, estimated GFR; GFR, glomerular filtration rate; mGFR, measured GFR.

In Figure 2, we summarize the performance of eGFR slope_cr-batch_, eGFR slope_cys_, and eGFR slope_cr-cys-batch_, showing that the combination of creatinine and cystatin C correlated better with mGFR slope than either filtration marker alone. In addition, the Bland-Altman plot for eGFR_cr-cys_ showed a lower margin for the limits of agreement in comparison with eGFR_cr_ and eGFR_cys_, indicating improved precision (Figure 2 panels b1–b3).

The correlations between mGFR slope, eGFR_cr_, eGFR_cys_, and eGFR_cr-cys_ separately for patients with 2, 3, and 4 mGFR measurements (corresponding to 3, 6, and 9 years of follow-up) are shown in Supplementary Table S3. eGFR_cr-cys_ slopes generally correlated better with mGFR slopes than eGFR_cr_ and eGFR_cys_, with the difference in correlations being more pronounced at shorter follow-up durations.

### eGFR Slope_cr-routine-annual_ Versus eGFR Slope_cr-routine-corresponding_

The correlations between eGFR slope_cr-routine-annual_ or eGFR slope_cr-routine-corresponding_ and mGFR slope during follow-up are displayed in Table 4. The correlation between eGFR slope_cr-routine-annual_ and mGFR slope (CCC: 0.49 [95% CI: 0.37–0.59]) was similar to that of eGFR slope_cr-routine-corresponding_ (*P* = 0.78).

### Sample Size Calculations

**Figure 3:**
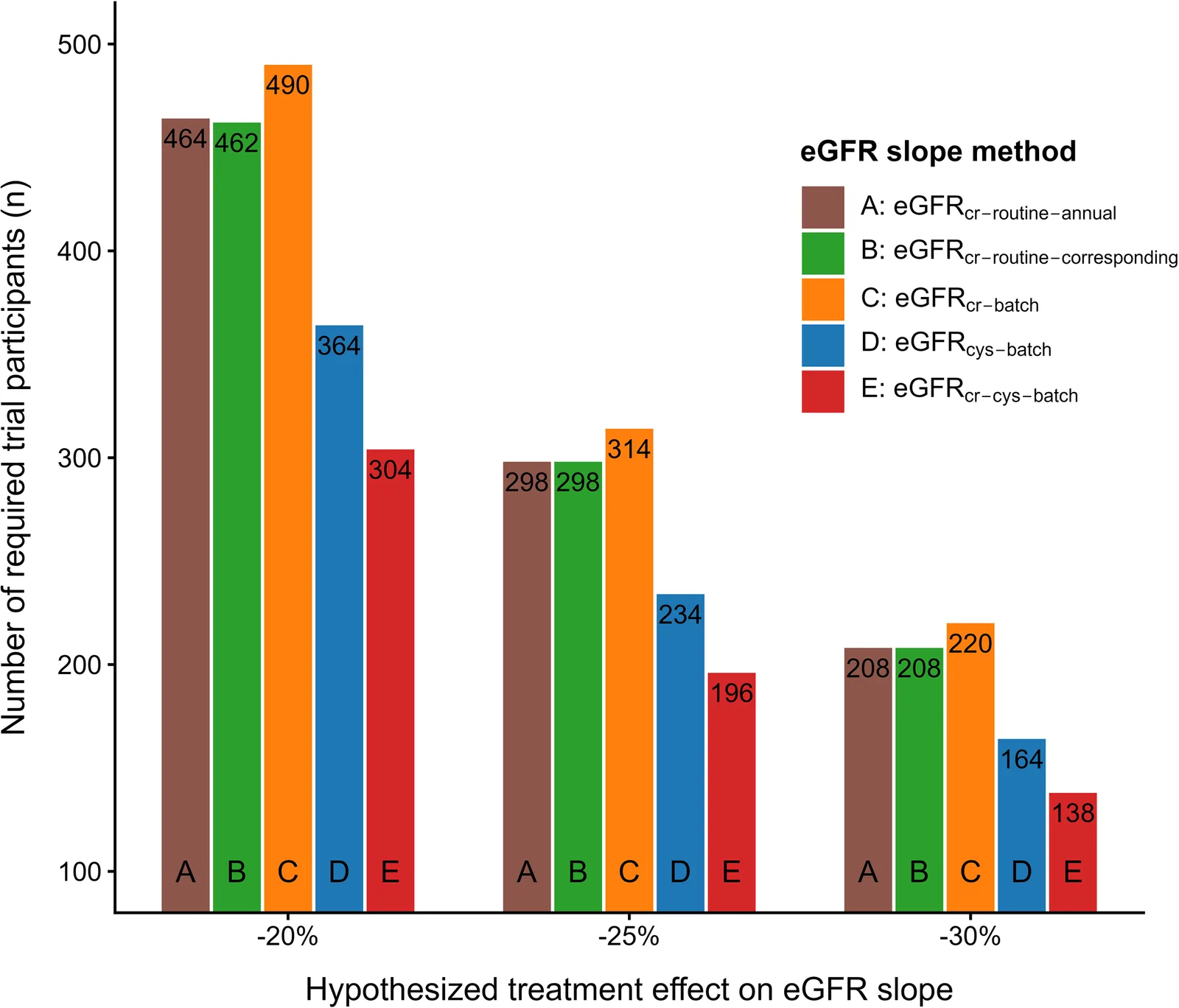
Sample size calculations by eGFR estimation method and hypothesized treatment effect, assuming a typical 2-arm randomized controlled trial design with 80% power, a significance level (α) of 0.05, 1:1 enrolment, and 20% dropout rate. Numbers represent the total number of participants required for the trial. eGFR, estimated glomerular filtration rate.

In the 99 patients typically enrolled in clinical ADPKD trials (aged ≤ 55 years, Mayo Imaging Classification 1C/1D/1E, and GFR ≥ 30 ml/min per 1.73 m^2^), the mean ± SD slopes of eGFR_cr-routine-annual_, eGFR_cr-routine-corresponding_, eGFR_cr-batch_, eGFR_cys-batch_, and eGFR_cr-cys-batch_ during the first 3 years were −3.81 ± 2.61, −4.65 ± 3.18, −4.43 ± 3.12, −4.11 ± 2.49, and −4.39 ± 2.43 ml/min per 1.73 m^2^/yr, respectively. Notably, slope SDs were relatively low for eGFR slope_cys-batch_ and eGFR slope_cr-cys-batch_. An overview of sample size calculations for each GFR slope method in a hypothetical 2-arm trial is presented in Figure 3. The required sample sizes were substantially lower for eGFR_cr-cys_–based slope methods than for eGFR_cr_, largely due to reduced variability in eGFR slope estimates. For example, assuming a treatment effect corresponding to a 20% reduction in eGFR slope, the estimated sample size was 304 participants for eGFR_cr-cys_ versus 490 for eGFR_cr_, representing a 38% reduction. Assuming treatment effects of 25% and 30% reductions in eGFR slope, eGFR_cr-cys_–based methods required 38% and 37% fewer participants, respectively, compared with eGFR_cr_. Overall, these findings suggest that trials using eGFR_cr-cys_–based end points may achieve comparable statistical power with substantially smaller sample sizes than trials using either creatinine- or cystatin C alone.

## Discussion

In clinical trials for ADPKD, assessment of the rate of GFR decline is the primary surrogate end point for evaluating new treatments, highlighting the importance of accurate and reliable methods to measure change in eGFR over time. The combined use of creatinine and cystatin C to estimate GFR enhanced precision and outperformed using either filtration marker alone in tracking mGFR slope, reflecting superior reliability for longitudinal monitoring. The use of creatinine-cystatin C–based eGFR reduced variability in slope estimates, thereby lowering sample size requirements for clinical trials. We found similar performance for batch-measured creatinine to estimate GFR compared with routine creatinine measurements obtained over several years. These findings support integrating cystatin C for GFR estimation to enhance trial efficiency in ADPKD.

The enhanced precision to estimate GFR for eGFR equations using both creatinine and cystatin compared with those using only 1 filtration marker was described originally at the publication of the 2012 CKD-EPI equation.^19^ These findings were later supported by Stehlé *et al.*^11^ in a recent review, which proposed that the combined use of creatinine and cystatin C helps to mitigate the influence of fluctuations in each of these biomarkers owing to non-GFR determinants. Consistent with these studies, our analysis indicated that creatinine-cystatin C–based eGFR equations exhibited the highest precision and the lowest SD in eGFR slope within a hypothetical trial population, leading to lower sample size requirements. Although our findings are not necessarily specific to ADPKD and likely also apply to other forms of CKD, they are particularly relevant to the ADPKD community, where achieving adequate statistical power is challenging because of the disease’s rarity compared with more prevalent conditions such as diabetes mellitus and hypertension. In addition, ADPKD trials may be more susceptible to fluctuations in serum creatinine, because patients are generally younger and more physically active than those with other kidney diseases, potentially leading to greater variability in creatinine levels. Furthermore, earlier studies have reported alterations in the tubular secretion of creatinine in patients with ADPKD,^27^ which may affect the accuracy of creatinine-based eGFR in this population.

A recent study reported that cystatin C–based eGFR performed worse than creatinine-based eGFR to assess change in kidney function over time in patients with ADPKD.^28^ In contrast, our findings demonstrate that the correlation between cystatin C–based eGFR slope and mGFR slope was comparable with that of creatinine-based eGFR slope. This discrepancy could be attributed to several factors: the previous study had a smaller number of patients with available cystatin C measurements (*n* = 97 vs. 260), a shorter follow-up period, and used a different mGFR method (iohexol plasma clearance vs. iothalamate urinary clearance). In addition, variability in cystatin C assays over time could contribute, because measurements in the previous study were performed at multiple time points rather than in a single batch, as in our study.

mGFR is considered the gold standard for assessing kidney function. However, frequent mGFR measurements are not feasible in clinical care or trials because of their cost, invasiveness, and logistical burden.^10^^,^^11^ In practice, this may lead to underpowered studies or trials that are difficult to conduct because of high costs, thus raising the risk of type 2 errors, that is, failing to detect a treatment effect when one exists. Our findings suggest that statistical power can be improved by using eGFR based on combined creatinine–cystatin C. Compared with eGFR_cr_, eGFR_cr-cys_ achieved comparable power to detect treatment effects while requiring approximately 37% to 38% fewer participants. These reductions translate to proportional cost savings, which is helpful for investigator-initiated and industry-driven trials, and help accelerate the execution of clinical trials in ADPKD. For example, in our investigator-initiated HYDRO-PROTECT trial, which evaluates the coprescription of hydrochlorothiazide alongside tolvaptan, a sample size reduction of 37% could have avoided the need to open 8 additional trial sites, corresponding to an estimated cost saving of approximately 30% of the total trial budget. These considerations are especially important for trials that might struggle to reach inclusion numbers, such as smaller investigator-initiated trials, or because they focus on a subgroup of patients with ADPKD, like those treated with tolvaptan or who have specific genotypes.^29^^,^^30^

The DIPAK observational study investigates disease progression in patients with ADPKD. Patients with concomitant diseases that affect kidney function, such as diabetes mellitus, are excluded from participation. This may affect the generalizability of our results for real-world patients. However, clinical trials in ADPKD generally exclude patients with other kidney diseases. Therefore, we believe this may improve the comparability of our findings to typical trial populations, although some differences may remain.

Our study has limitations. As in nearly all observational studies, routine cystatin C measurements were not available, so we could not compare routine to batch-measured cystatin C. This comparison would have been relevant because cystatin C measurements were previously shown to be influenced by drift and day-to-day variation, despite standardized laboratory practices.^14^ In addition, because of costs and the burden on patients, mGFR was not measured annually. In real clinical trials, mGFR would likely be available at selected time points, and mGFR decline measured over a 3-year period (the typical duration for ADPKD trials). Therefore, we chose to retain patients with 3 years of follow-up in the analyses. For the sample size calculations, we selected patient populations that approximate those typically included in clinical trials. However, we cannot account for all possible trial-specific inclusion and exclusion criteria, which may lead to differences in patient characteristics and could influence the generalizability of our findings. In addition, data on thyroid function, which can affect cystatin C levels, were not available. The strengths of our study are that we included comparisons with mGFR, the single-batch remeasurement of large numbers of samples to mimic clinical trial settings, and the use of a well-phenotyped cohort. In addition, in contrast to most previous studies that compared creatinine- to cystatin-based eGFR in ADPKD, this study particularly focused on the consequences for trial design, which is an important aspect of advancing treatment for ADPKD.

In conclusion, incorporating cystatin C enhanced precision when combined with creatinine. The use of creatinine-cystatin C–based estimates reduce eGFR slope variability, lowering sample size requirements for clinical trials. Based on our findings, we recommend using creatinine-cystatin C–based eGFR in ADPKD clinical trials to optimize the detection of treatment effects on kidney function.

## Disclosure

All the authors declare no competing interests.

## Supplementary Material

Supplementary File (PDF) **Supplementary Methods:** Description of measured GFR methodology. **Supplementary Data:** Analytical validity of single-batch measurement. **Figure S1.** Subgroup analysis for eGFR slope bias using routine plasma creatinine values (CKD-EPI 2021). **Figure S2.** Subgroup analysis for eGFR slope bias using batch-measured plasma creatinine values (CKD-EPI 2021). **Figure S3.** Cross-sectional and longitudinal performance of eGFR_cys-batch_ (CKD-EPI 2012) and eGFR_cr-cys-batch_ (CKD-EPI 2021) for mGFR assessment. **Figure S4.** Subgroup analysis for eGFR slope bias using batch-measured plasma cystatin values (CKD-EPI 2012). **Figure S5.** Subgroup analysis for eGFR slope bias using batch-measured plasma creatinine and cystatin values (CKD-EPI 2021).**Table S1.** Cross-sectional performance of alternative eGFR equations. **Table S2.** Correlations between eGFR slope (CKD-EPI 2009 and EKFC equations) and mGFR slope. **Table S3.** Summary of concordance correlation coefficients between mGFR slope and eGFR slope at various time points.

## Footnotes

Supplementary File (PDF)
**Supplementary Methods:** Description of measured GFR methodology. **Supplementary Data:** Analytical validity of single-batch measurement. **Figure S1.** Subgroup analysis for eGFR slope bias using routine plasma creatinine values (CKD-EPI 2021). **Figure S2.** Subgroup analysis for eGFR slope bias using batch-measured plasma creatinine values (CKD-EPI 2021). **Figure S3.** Cross-sectional and longitudinal performance of eGFR_cys-batch_ (CKD-EPI 2012) and eGFR_cr-cys-batch_ (CKD-EPI 2021) for mGFR assessment. **Figure S4.** Subgroup analysis for eGFR slope bias using batch-measured plasma cystatin values (CKD-EPI 2012). **Figure S5.** Subgroup analysis for eGFR slope bias using batch-measured plasma creatinine and cystatin values (CKD-EPI 2021). **Table S1.** Cross-sectional performance of alternative eGFR equations. **Table S2.** Correlations between eGFR slope (CKD-EPI 2009 and EKFC equations) and mGFR slope. **Table S3.** Summary of concordance correlation coefficients between mGFR slope and eGFR slope at various time points.
