# Supplementary material for: Combining Cystatin C and Creatinine Increases eGFR Slope Accuracy in ADPKD
Source: Kidney Int Rep. 2026 Aug 6;11(10):107008. doi: 10.1016/j.ekir.2026.107008 (PMC13571460; doi:10.1016/j.ekir.2026.107008)

Supplementary Material

Table of contents

1. Supplementary Methods: Description of measured GFR methodology.

2. Supplementary data: Analytical validity of single-batch measurement

3. Supplementary Table 1: Cross-sectional performance of alternative eGFR equations.

4. Supplementary Table 2: Correlations between eGFR slope (CKD-EPI 2009 and EKFC equations) and mGFR slope.

5. Supplementary Table 3. Summary of concordance correlation coefficients between mGFR slope and eGFR slope at various timepoints.

6. Supplementary Figure 1: Subgroup analysis for eGFR slope bias using routine plasma creatinine values (CKD-EPI 2021).

7. Supplementary Figure 2: Subgroup analysis for eGFR slope bias using batch-measured plasma creatinine values (CKD-EPI 2021).

8. Supplementary Figure 3: Cross-sectional and longitudinal performance of $eGFR_{cys\text{-}batch}$ (CKD-EPI 2012) and $eGFR_{cr\text{-}cys\text{-}batch}$ (CKD-EPI 2021) for mGFR assessment.

9. Supplementary Figure 4: Subgroup analysis for eGFR slope bias using batch-measured plasma cystatin values (CKD-EPI 2012).

10. Supplementary Figure 5: Subgroup analysis for eGFR slope bias using batch-measured plasma creatinine and cystatin values (CKD-EPI 2021).

Supplementary Methods. Description of measured GFR methodology

After a stabilization period of 90 minutes, two urine clearance periods of 120 minutes each were conducted. During these periods, naturally voided urine was collected. Blood samples were drawn at 90, 150, 210, 270, and 330 minutes to ensure accurate monitoring of serum concentrations of the tracers. Following the collection of a pre-dose blood sample, a priming dose was administered intravenously containing 0.3 MBq of ^{125}I-iothalamate (Glofil-125, Iso-Tex Diagnostics, USA) and 0.4 MBq of ^{131}I-hippuran (Hippuran, Polatom, Poland). This was followed by a continuous infusion of these tracers at a concentrations of 0.0075 MBq/mL ^{125}I-iothalamate and 0.02 MBq/mL ^{131}I-hippuran at a rate of 9 mL/h or 12 mL/h, depending on the serum creatinine level, over a period of 330 minutes. Radioactivity levels of ^{125}I-iothalamate and ^{131}I-hippuran in serum, urine, and infusion solution were measured using a Perkin Elmer Wizard 2470 gamma counter (Waltham, MA, USA).

Following a 1.5-hour equilibration period to achieve stable serum concentrations of both tracers, urinary clearance was measured simultaneously during two consecutive 2-hour periods. The urinary clearance of ^{125}I-iothalamate and ^{131}I-hippuran was calculated as:

$$\text{Urinary clearance} = \frac{\text{U} * \text{V}}{\text{P}}$$

where U × V represents urinary excretion of the tracer and P represents radioactivity levels in serum. The serum clearance was calculated as:

$$\text{Serum clearance} = \frac{\text{I} * \text{V}}{\text{P}}$$

where I × V represents the infusion rate of the filtration marker at steady state, and P represents the serum radioactivity levels. Given that ^{131}I-hippuran clearance occurs exclusively through renal elimination, its serum clearance equals urinary clearance under steady-state conditions. The urinary clearance of ^{125}I-iothalamate was corrected for voiding errors using the serum-to-urinary clearance ratio of ^{131}I-hippuran.

Supplementary data: Analytical validity of single-batch measurement

All routine measurements and single-batch remeasurements were performed in our laboratory which performs Internal quality controls (IQC) measurements and participates in external quality assessment (EQA) programs. IQC were performed using the Unassayed Chemistry Plus (creatinine) and the Cystatin C Control Set Gen.2 (cystatin C) reagents.

The results for IQC are as follows:

For creatinine, analytical performance was as follows: at the low QC level (~62 μmol/L), within-run CV, between-run CV, total CV, and bias were 1.29%, 0.74%, 1.49%, and -4.01%, respectively; at the high QC level (~636 μmol/L), these were 0.56%, 0.00%, 0.54%, and -1.72%, respectively. For cystatin C, at a low QC level (~0.94 mg/L), within-run CV, between-run CV, total CV, and bias were 1.34%, 0.50%, 1.43%, and 5.97%, respectively; at a high QC level (~4.84 mg/L), these were 0.44%, 0.05%, 0.45%, and 2.85%, respectively. The observed within-run CVs were below the manufacturer-reported maximum imprecision (creatinine: 2.6% and 2.0%; cystatin C: 4.0% at low and high concentration levels respectively). The routine EQA and the single batch measurements for creatinine and cystatin C had similar CVs.

Biobanked serum samples were stored at -80°C for 0.46 to 11.54 years (median 7.22 years, with a broad distribution across the storage interval). Biobanked samples underwent a single freeze-thaw cycle. Batch measurements were performed using the same reagent lot.

Supplementary Table 1. Cross-sectional performance of the 2021 CKD EPI and EKFC equations. 95% Confidence intervals are given in brackets.

Variable	Bias (ml/min/1.73m^2)	Precision (ml/min/1.73m^2)	P30 (%)	P10 (%)	Agreement (%)
$eGFR_{cr-batch}$ (CKD-EPI 2009)	-0.8 [-1.9, 0.4]	9.0 [7.9, 10.1]	96.5 [93.9, 98.7]	61.0 [54.5, 67.5]	74.9 [69.3, 80.1]
$eGFR_{cr-cys-batch}$ (CKD-EPI 2012)	-3.9 [-4.9, -3.0]	7.4 [6.5, 8.4]	97.8 [95.7, 99.6]	54.5 [48.1, 60.6]	73.6 [68.0, 79.7]
$eGFR_{cr-batch}$ (EKFC 2021)	-2.9 [-3.9, -1.9]	8.1 [7.2, 8.9]	98.7 [97.0, 100.0]	61.5 [55.4, 67.5]	74.5 [68.4, 79.7]
$eGFR_{cys-batch}$ (EKFC 2023)	-2.6 [-3.8, -1.5]	9.2 [8.3, 10.1]	95.7 [92.6, 98.3]	51.1 [44.2, 57.6]	66.2 [60.2, 71.9]
$eGFR_{cr-cys-batch}$ (EKFC 2023)	-2.7 [-3.7, -1.8]	7.3 [6.5, 8]	97.4 [95.2, 99.1]	64.5 [58.4, 70.6]	77.1 [71.9, 82.3]

CKD-EPI: Chronic Kidney Disease Epidemiology Collaboration*;* eGFR: estimated glomerular filtration rate, EKFC: European Kidney Function Consortium; mGFR: measured glomerular filtration rate

Supplementary Table 2. Summary of eGFR slopes for the alternative CKD-EPI and EKFC equations, with CCC estimates for the correlation with mGFR slope.

Variable	Slope ± SD (ml/min/1.73m^2/year)	CCC [95% CI]
$eGFR_{cr\text{-}batch}$ (CKD-EPI 2009)	-3.33 ± 2.27	0.58 [0.47, 0.67]
$eGFR_{cr\text{-}cys\text{-}batch}$ (CKD-EPI 2012)	-3.13 ± 1.94	0.68 [0.58, 0.75]
$eGFR_{cr\text{-}batch}$ (EKFC 2021)	-3.11 ± 2.15	0.62 [0.52, 0.71]
$eGFR_{cys\text{-}batch}$ (EKFC 2023)	-2.69 ± 1.92	0.63 [0.53, 0.71]
$eGFR_{cr\text{-}cys\text{-}batch}$ (EKFC 2023)	-2.90 ± 1.84	0.70 [0.61, 0.77]

CCC: concordance correlation coefficient; CKD-EPI: Chronic Kidney Disease Epidemiology Collaboration; eGFR: estimated glomerular filtration rate, EKFC: European Kidney Function Consortium; mGFR: measured glomerular filtration rate

Supplementary Table 3. Summary of concordance correlation coefficients between mGFR slope and eGFR slope for 2021 ($eGFR_{cr}$ and $eGFR_{cr\text{-}cys}$) and 2012 ($eGFR_{cys}$) CKD EPI equations, between baseline and various timepoints. Years 3, 6 and 9 represent patients with 2, 3 and 4 mGFR measurements, respectively. Confidence intervals (95%) are given in brackets.

Variable	Year 3	Year 6	Year 9
$eGFR_{cr\text{-}batch}$ (CKD-EPI 2021)	0.45 [-0.01, 0.76]	0.68 [0.47, 0.81]	0.66 [0.42, 0.82]
$eGFR_{cys\text{-}batch}$ (CKD-EPI 2012)	0.52 [0.33, 0.67]	0.74 [0.56, 0.86]	0.72 [0.51, 0.85]
$eGFR_{cr\text{-}cys\text{-}batch}$ (CKD-EPI 2021)	0.62 [0.18, 0.85]	0.77 [0.60, 0.87]	0.74 [0.55, 0.86]

CKD-EPI: Chronic Kidney Disease Epidemiology Collaboration*;* eGFR: estimated glomerular filtration rate,

Supplementary Figure 1. Subgroup analysis for slope bias ($eGFR_{cr\text{-}routine\text{-}corresponding}$ – mGFR) using the CKD-EPI 2021 equation. BMI: body mass index, CKD: chronic kidney disease; hsCRP: high sensitivity C-reactive protein; HtTKV: height-adjusted total kidney volume; uACR: urinary albumin/creatinine ratio

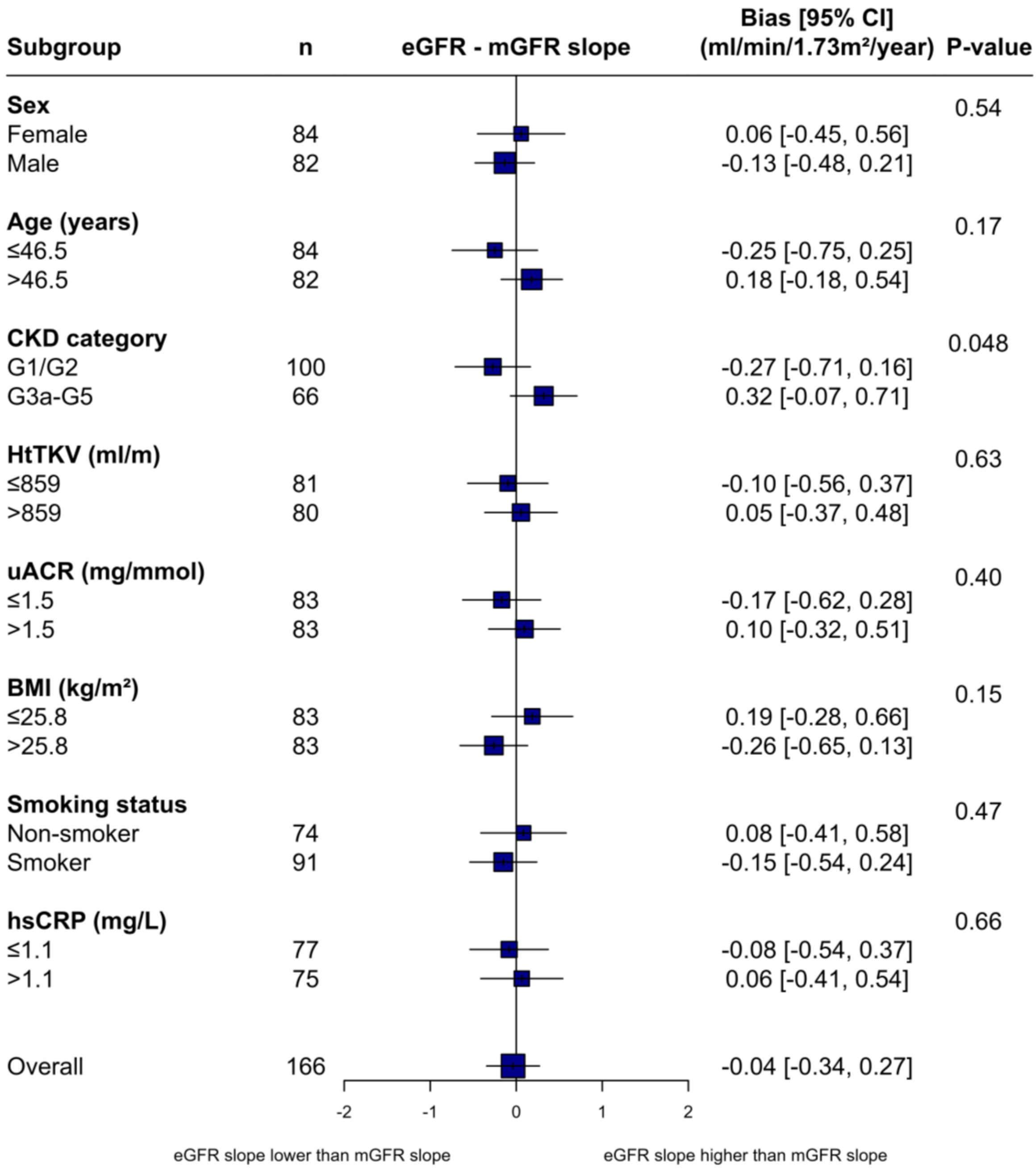

Supplementary Figure 2. Subgroup analysis for slope bias ($eGFR_{cr\text{-}batch}$ – mGFR) using the CKD-EPI 2021 equation. BMI: body mass index, CKD: chronic kidney disease; hsCRP: high sensitivity C-reactive protein; HtTKV: height-adjusted total kidney volume; uACR: urinary albumin/creatinine ratio

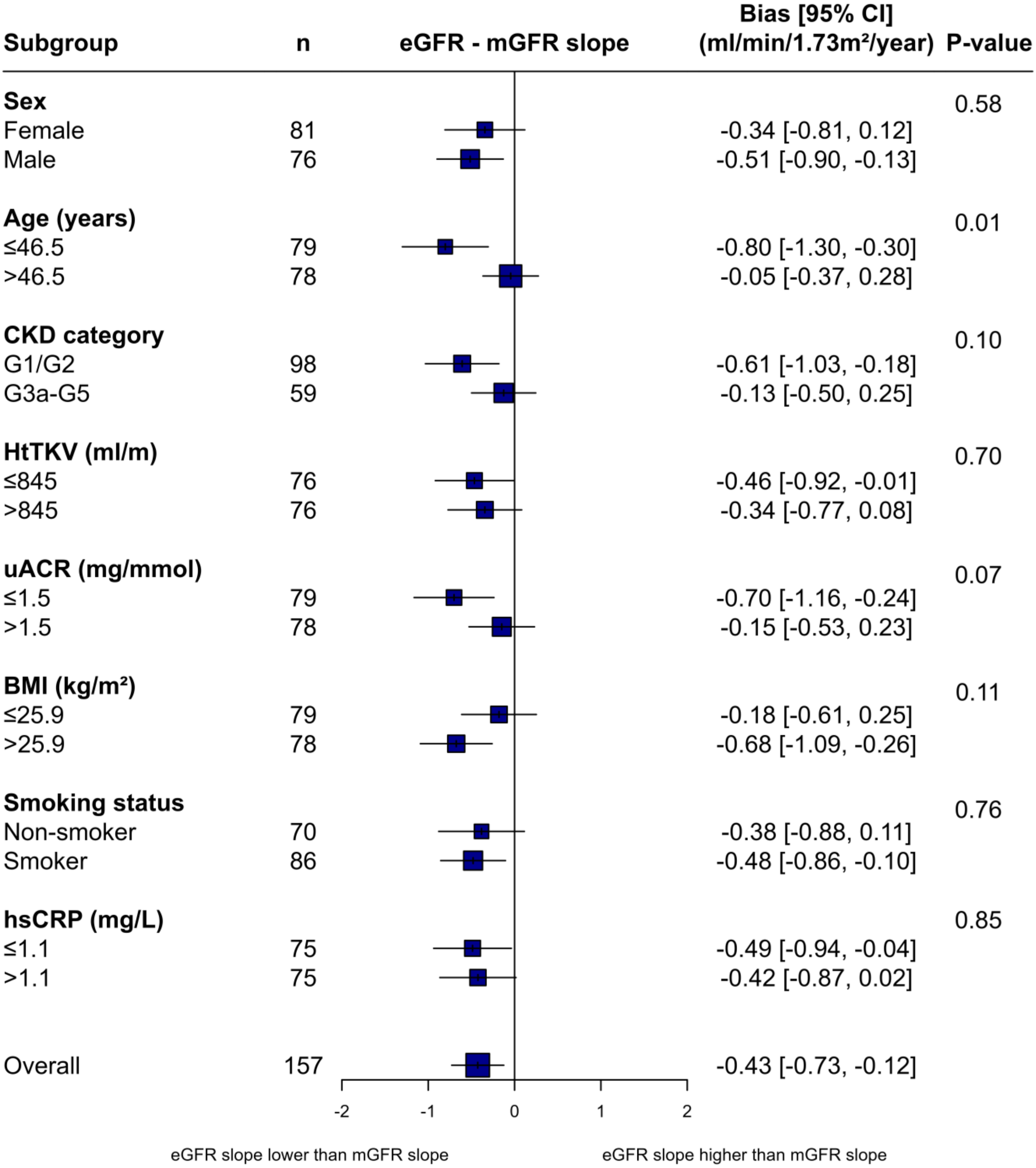

Supplementary Figure 3. eGFR performance (batch-measured cystatin-based CKD-EPI 2012 and batch-measured creatinine-cystatin C-based CKD EPI 2021) for cross-sectional mGFR assessment (A) and longitudinal GFR slope (B/C). **A**: Solid black line = identity; dotted red lines = P30 (±30%); gray dashed lines = CKD stage thresholds; green line = LOESS regression. Mean bias ± precision (ml/min/1.73m²) shown. **B**: Dotted line = orthogonal regression; solid line = identity. **C**: Black dotted line = mean bias (eGFR – mGFR slope); red dotted lines = limits of agreement. CCC = concordance correlation coefficient; CI = confidence interval; SD = standard deviation.

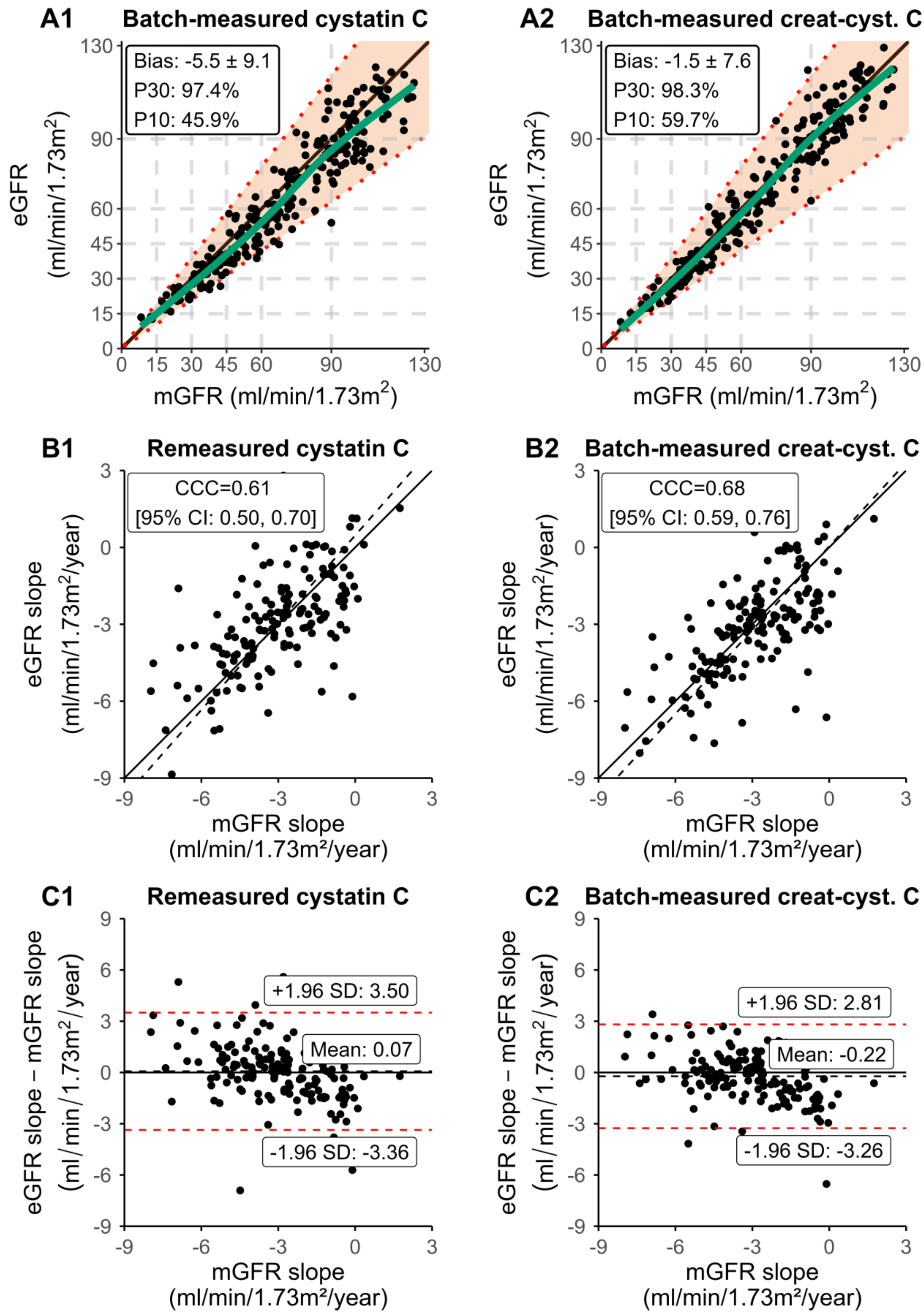

Supplementary Figure 4. Subgroup analysis for slope bias ($eGFR_{cys\text{-}batch}$ – mGFR) using the CKD-EPI 2012 equation. BMI: body mass index, CKD: chronic kidney disease; hsCRP: high sensitivity C-reactive protein; HtTKV: height-adjusted total kidney volume; uACR: urinary albumin/creatinine ratio

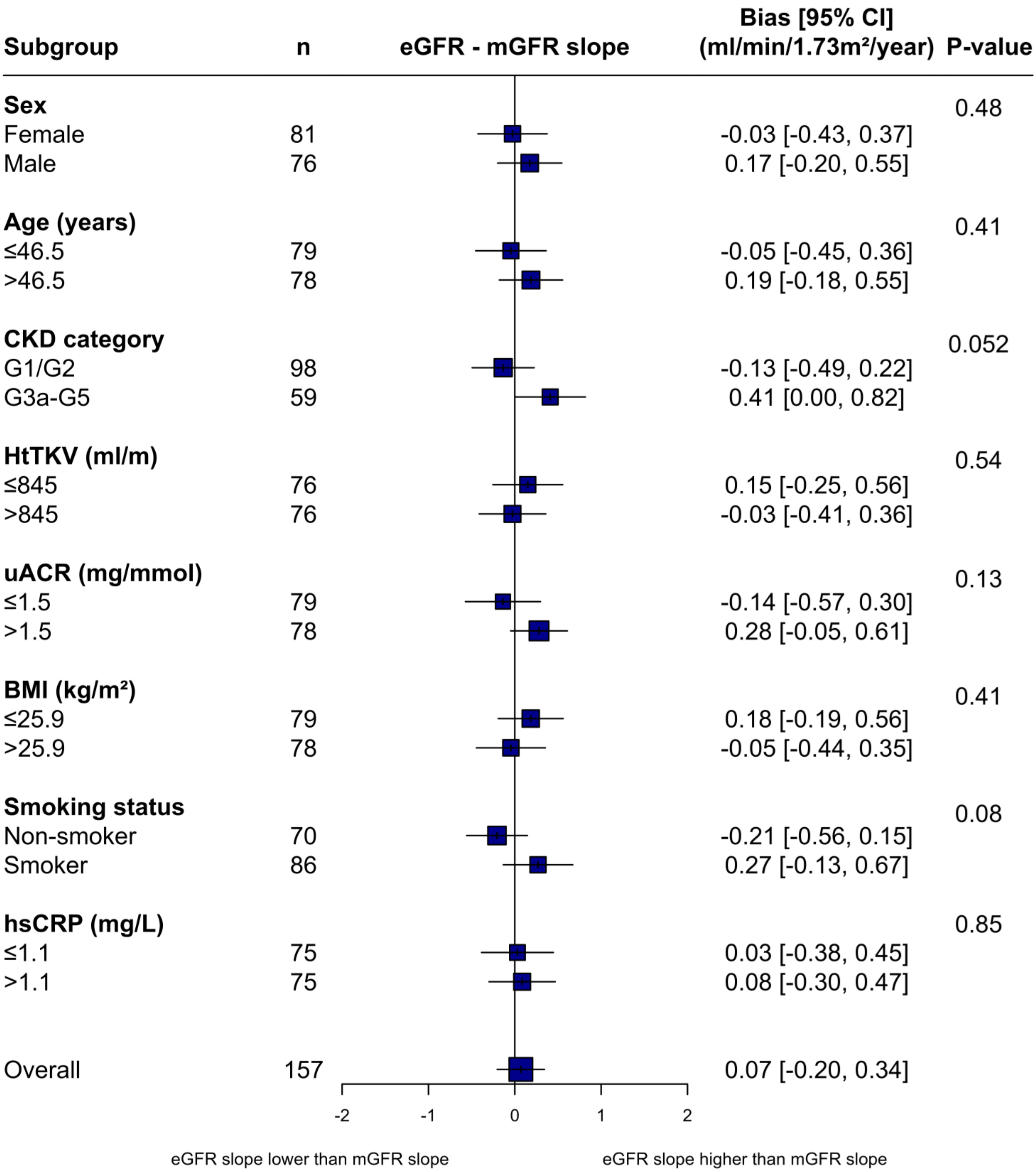

Supplementary Figure 5. Subgroup analysis for slope bias ($eGFR_{cr-cys-batch}$ – mGFR) using the CKD-EPI 2021 equation. BMI: body mass index, CKD: chronic kidney disease; hsCRP: high sensitivity C-reactive protein; HtTKV: height-adjusted total kidney volume; uACR: urinary albumin/creatinine ratio

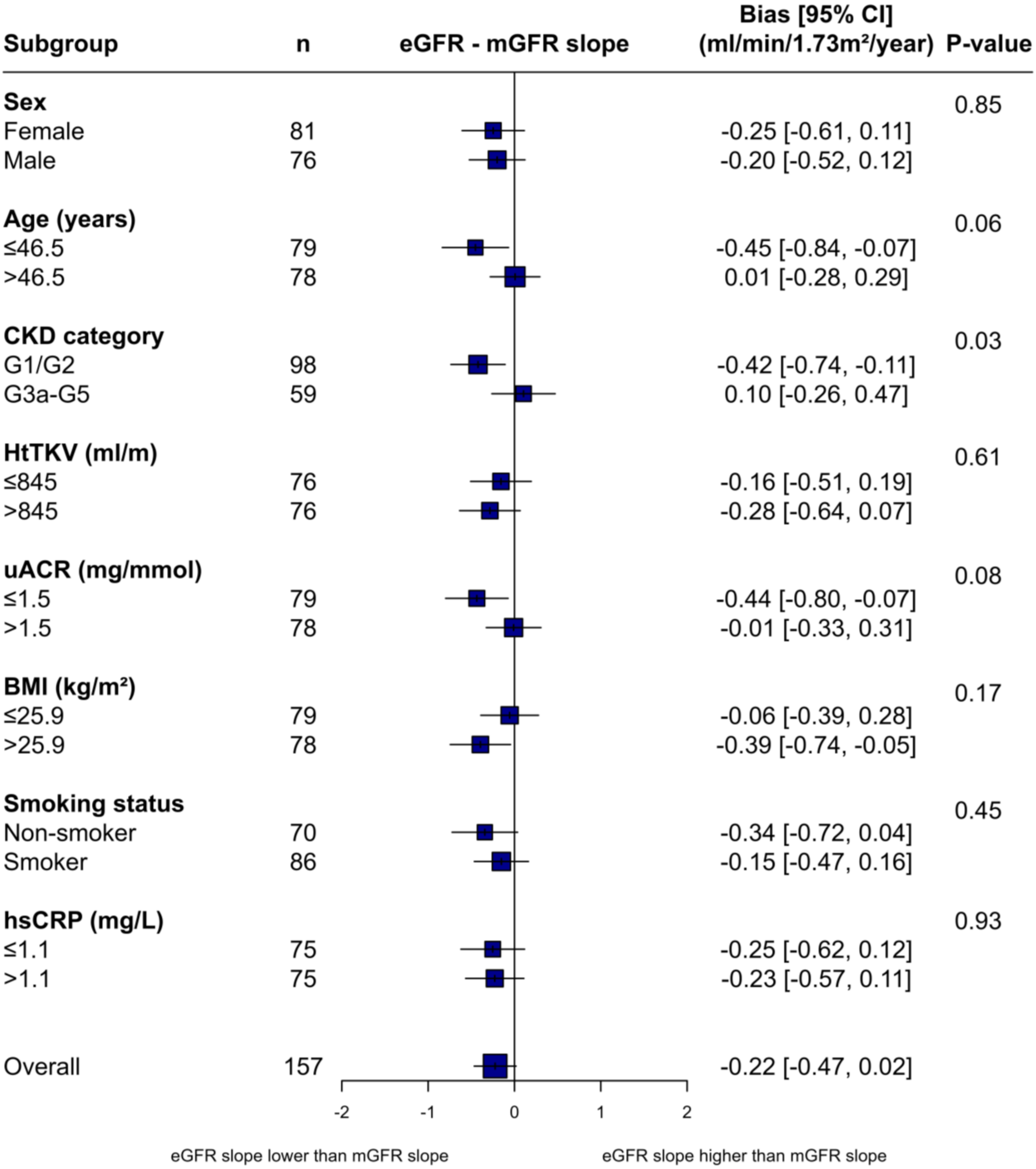

Supplement: Supplementary file 1 [file mmc1.pdf]
